# Heterocyclic biradicaloid for singlet fission: cleavage of bromine atoms from precursor 3,6-dibromo-1,4-dimethyl-piperazine-2,5-dione†

**DOI:** 10.1039/d5ra07891a

**Published:** 2025-12-16

**Authors:** Paul Dron, Radek Pohl, Josef Cvačka, Miroslav Dudič, František Vavrek, Lubomír Pospíšil

**Affiliations:** a Institute of Organic Chemistry and Biochemistry of the Czech Academy of Sciences Flemingovo nám. 2 Prague 160 00 Czech Republic lubomiir.pospisil@@jh-inst.cas.cz; b J. Heyrovský Institute of Physical Chemistry of the Czech Republic Dolejškova 3 Prague 182 23 Czech Republic

## Abstract

Singlet fission is a spin-allowed process, unique to molecular photophysics, whereby one singlet excited state is converted into two triplet states. The phenomenon has been observed in molecular crystals, aggregates, disordered thin films, and covalently-linked dimers. The chromophores are oriented such that the electronic coupling between the singlet and the double triplet states is large. A compound of 3,6- dibromo-1,4-dimethyl-piperazine-2,5-dione is a precursor of a dication, which is expected to yield the singlet fission effect. The reversible reductive cleavage of two C–Br bonds at −0.4 V was observed using a mercury electrode. Unexpectedly, mercury reacts by adsorptive cleavage of C–Br bonds, yielding the target product and Hg_2_Br_2_. This is a significant simplification of the preparation protocol. Voltammetry on a glassy carbon electrode showed an irreversible cleavage at ∼−1.2 V without any sign of adsorption. Spontaneous adsorptive cleavage of C–Br bonds confirms the role of the electrode material. Mechanism was confirmed by the time dependence of UV-Vis spectra, NMR technique of H,C-HMBC, and MS products analysis. The target product is highly reactive.

## Abstract 2

Singlet fission is a spin-allowed process, unique to molecular photophysics, whereby one singlet excited state is converted into two triplet states.

## Graphical abstract

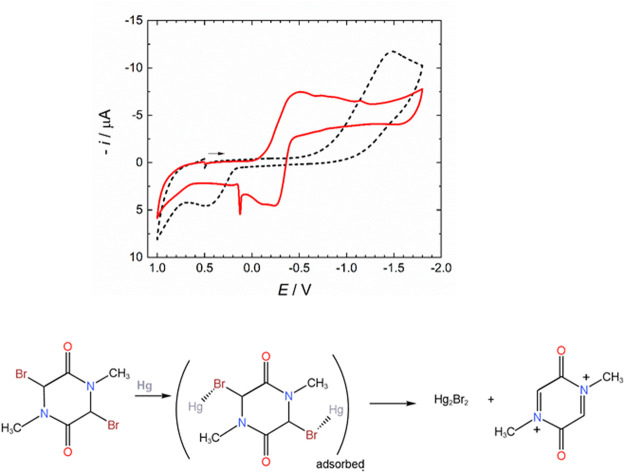

## 1 Introduction

Singlet fission (SF) is a photophysical reaction typical of some organic compounds where an excited singlet state yields two spin-triplets.^1,2^ The effect leads to the production of two electrons instead of a single electron. Efficiency is of interest for many laboratories. Singlet fission is the subject of several theoretical reviews.^3–6^ A suitable compound has to fulfil several criteria. Crystal structure and molecular packing influence the performance. Efficient singlet fission requires a singlet state energy (E(S_1_)) at least twice the triplet state energy (E(T_1_)): E(S_1_) ≥ 2 × E(T_1_). Suitable materials include acenes (*e.g.*, tetracene, pentacene), perylene derivatives, and diketopyrrolopyrroles. The preparation of 2 has apparently never been attempted. An adduct of a hydrogen atom to 2 was generated by hydrogen atom abstraction using a pulse radiolytic method and the radical was investigated as a short-lived transient.^7^ Recently, intramolecular SF (iSF) has been reported in covalently linked molecular oligomers. Unique advantages is the iSF mechanism and straightforward practical applications. Achieving efficient iSF in endothermic/isothermal systems is necessary. Unfortunately, the design strategy for efficient iSF based on endothermic/isothermal chromophores is not clear.^8^ A compound having an inefficient SF property was recently activated using proper substitutions.^9^ This communication will report on a surprisingly simple protocol based on electrochemical cleavage of C–Br bonds, taking advantage of mercury electrodes over all other types of electrode materials.

**Scheme 1:**
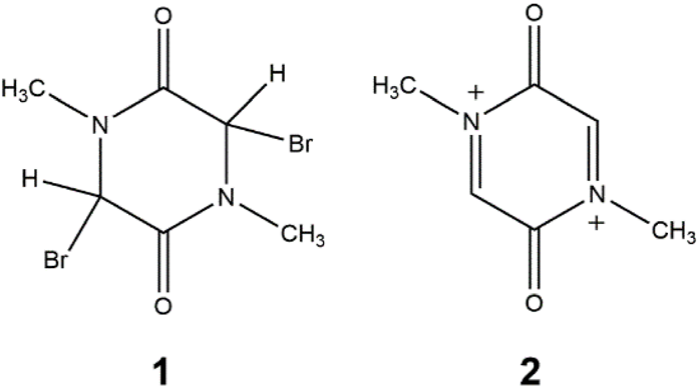
Starting compound 1 and the target compound 2.

The electrochemical cleavage of carbon–halogen bonds in organic halogen derivatives has been studied in numerous previous reports^10,11^ The mechanism of the electron transfer and bond cleavage may involve either the concerted or the stepwise mechanism.^12,13^ Radical intermediates often undergo dimerization, forming the dimer, which readily decomposes upon oxidation^14–17^ A compound of 3,6-dibromo-1,4-dimethyl-piperazine-2,5-dione (1) was synthesized. Electrochemical redox debromation of 1 to dication 2 (Scheme 1) should yield a biradicaloid with the singlet fission effect. This communication describes the unexpected spontaneous debromation that occurs when 1 comes into contact with metallic mercury. Hence, the theoretical prediction indicated that 2 could be a suitable candidate (Fig. S1) for the singlet fission effect.^1^

## 2 Experimental

### 2.1 Materials

Acetonitrile (CH_3_CN, 99.8%, anhydrous) was obtained from Sigma Aldrich, Germany, (5 mL). It was refluxed with CaH_2_ for 60 minutes and distilled off. The entire procedure was performed in argon atmosphere. The obtained solvent was kept under activated molecular sieve (3 Å) for 48 hours. Tetrabutylammonium hexafluorophosphate was p. a. grade for electrochemical use (TBAPF_6_, ≥99%, Sigma Aldrich, Switzerland). It was dried in the oven at 80 C. Argon gas (99.998%, Messer, Czech Republic) was used as received. Metallic mercury (Sigma Aldrich, Switzerland) was of electrochemical grade. Synthesis of 3,6-dibromo-1,4-dimethylpiperazine-2,5-dione was performed following the published in ref. 18 and sealed in ampules.

### 2.2 Electrochemical methodology

Electrochemical methods involved DC polarography, cyclic voltammetry (CV), exhaustive electrolysis, and UV-Vis spectroelectrochemistry. Concentration of molecules in experiments was typically 1 × 10^−3^ M. Electrochemical data were used with a fast solid-state potentiostat controlled by a computer *via* IEEE interface and data acquisition cards (PCL-848 and PCL-818, AdvanTech, USA) using 12-bit precision for A/D and D/A conversion. Exhaustive electrolysis was made using PGSTAT30 (Metrohm, Switzerland). Exhaustive electrolysis used a glassy carbon rod as a working electrode. A valve-operated and computer-controlled static mercury drop electrode SMDE (Laboratorní Přístroje Praha, Czech Republic) with an area of 0.0017 cm^2^ served as the working electrode for DC, AC polarography polarographic and CV experiments. The auxiliary electrode was a platinum wire. Glassy carbon electrode used in CV experiments had an area of 0.0058 cm2. The samples were prepared in dry acetonitrile (AN) using tetrabutylammonium hexafluorophosphate (TBAPF_6_) as an indifferent electrolyte. Samples were dried in vacuum in a Schlenk tube. The electrolyte TBAPF_6_ was dried the same way. The redox potential of the ferrocene/ferrocenium couple against our Ag|AgCl|1 M LiCl reference electrode was 0.56 V in acetonitrile. The numerical integration of the current *vs.* time decay yielded the transferred charge upon electrolysis. Spectra were measured in a 1 cm cuvette and the absorbance was divided by the concentration. During the voltage scan a diode-array UV-Vis spectrometer (Agilent, model 8453) collected spectra every 4 s. Typically, 600 spectra were collected.

### 2.3 Nuclear magnetic resonance

^1^H and ^13^C NMR spectra were acquired on a Bruker AVANCE IIIHD 500 (1H at 500.0 MHz, 13C at 125.7 MHz) spectrometer. ^1^H and ^13^C resonances were assigned using H,C-HMBC technique. All chemical shifts are quoted on the *δ* scale in ppm and referenced using residual ^1^H solvent signal in ^1^H NMR spectra (*δ*(CH_2_DCN) = 1.94 ppm) and ^13^C solvent signal in ^13^C NMR spectra (*δ*(CD_3_CN) = 1.32 ppm).

### 2.4 Mass spectrometry

The mass spectrum was recorded using an LTQ Orbitrap XL hybrid mass spectrometer (Thermo-Fisher Scientific, Waltham, USA) equipped with an electrospray ionization (ESI) source. The sample was introduced with a 5 µL injection loop into the mobile phase stream (methanol/water, 4 : 1, or acetonitrile) at a flow rate of 100 µL min^−1^. The spray voltage, capillary voltage, tube lens voltage and capillary temperature were set to 4.8 kV, 355 V, 145 V, and 275 °C, respectively.

## 3 Results and discussion

### 3.1 Electrochemistry

**Fig. 1:**
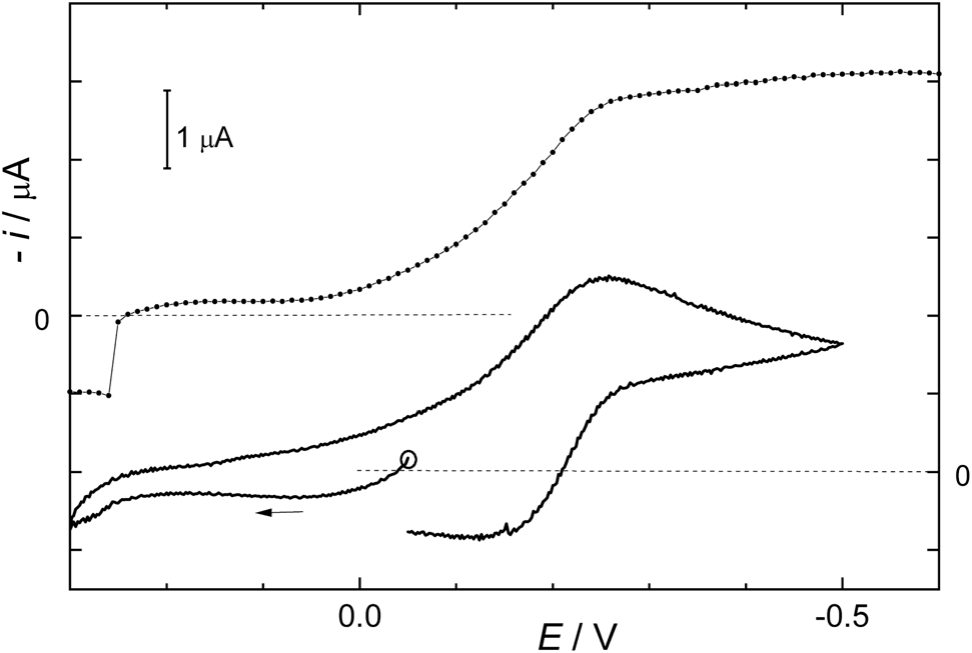
DC polarogram (upper curve) and cyclic voltammogram (lower curve) of 1 using a mercury drop electrode. The voltage scan was 0.1 V s^−1^, the solvent was acetonitrile with 0.1 M TBAPF_6_.

This section will show the differences between mercury electrodes and the glassy carbon electrode. The adsorption of 1 on Hg surface is essential for the cleavage of C–Br bonds. Redox properties of 1 measured by DC polarography or cyclic voltammetry using mercury electrodes are shown in Fig. 1. A reversible redox pair appears at 0.15 V. Furthermore, individual mercury droplets detached from the dropping mercury electrodes show a gray deposit and collect at the bottom of the electrochemical cell without coalescing in a mercury pool. This effect, rarely observed, points to the formation of an insoluble layer of Hg_2_Br_2_ at the surface of Hg droplets, preventing their conjoining. Strong adsorption of 1 at the mercury surface yields molecular bromine, keeping mercury droplets separated.

A test tube experiment under argon was performed, in which metallic mercury was added to the solution of 1 in acetonitrile (without electrochemical methods and a supporting electrolyte). The resulting sample changed from a colorless solution to a yellow one. Again, mercury separated into small, greyish droplets at the bottom of the test tube. Another test tube experiment using a diluted solution of Br_2_ in acetonitrile instead of Hg gave an identical outcome. Spectroscopy UV-Vis confirmed the release of bromine in solutions of 1 upon contacting added metallic mercury (see below).

**Fig. 2:**
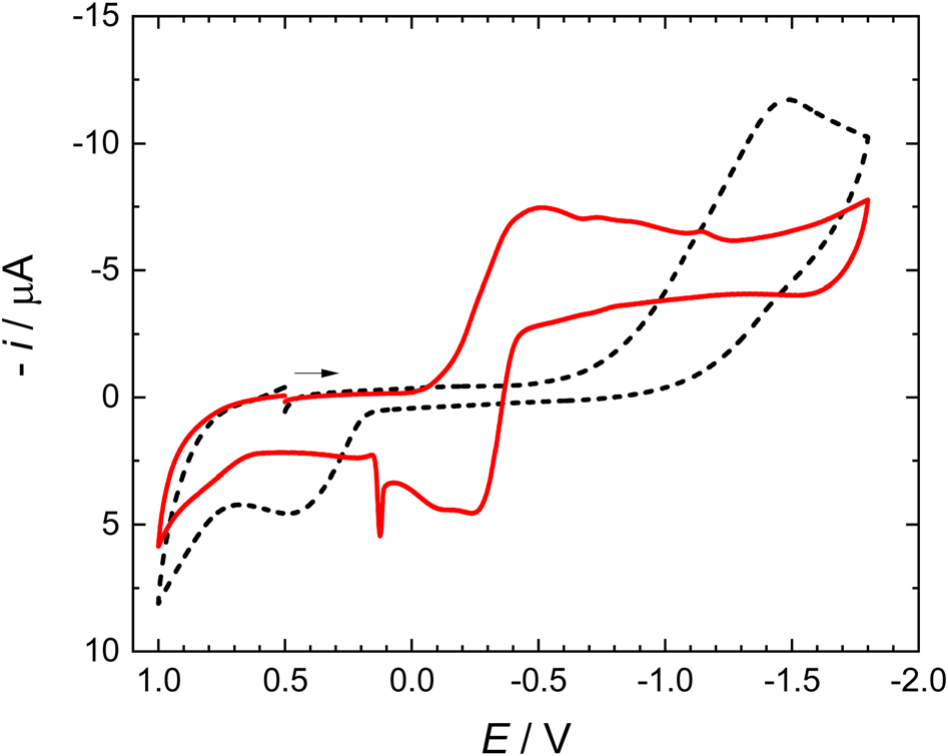
Cyclic voltammogram of 3.2 mM 1 and 0.1 M TBAPF_6_ in acetonitrile using a glassy carbon electrode (black dashed curve). Approximately 15 minutes after the addition of metallic mercury to the solution, a chemically reversible redox pair appears (red curve). The scan rate was 0.1 V s^−1^. A magnetic stirrer was used to stir the solution prior to measurements.

The working electrode for further experiments was the glassy carbon electrode. Cyclic voltammogram on glassy carbon electrode in the solution of 1 is given as a dashed curve in Fig. 2. It shows a reduction peak at −1.5 V and an oxidation current at +0.5 V. The irreversible reduction produces most likely a dimer, oxidized irreversibly at a positive potential. Such a dimerization mechanism is rather common for many organic redox systems.

**Scheme 2:**
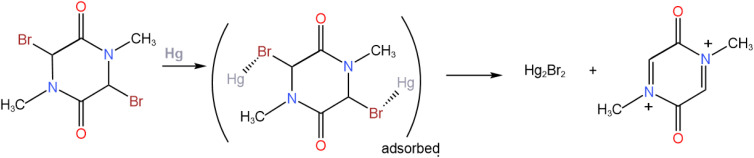
Adsorption of 1 on mercury drop working electrodes and also by injection of metallic mercury to the sample solution without applying the potential.

Using the glassy carbon working electrode, the addition of metallic mercury to the solution of 1 colorless solution turned it yellow. Voltammograms using the glassy carbon working electrode were registered at about 10-minutes intervals. They show a gradual decrease of a peak at −1.5 V and the development of a new pair of redox peaks; the final picture is the red curve in Fig. 2. It is evident that the presence of mercury initiates a spontaneous de-bromation of 1. Adsorption on mercury leads to the release of Br_2_, formation of Hg_2_Br_2,_ and appearance of a chemically reversible redox pair of peaks at −0.4 V. Mercury droplets do not join themselves because they are covered with the adsorbed Hg_2_Br_2_ layer (Scheme 2).

### 3.2 UV-Vis spectroscopy

**Fig. 3:**
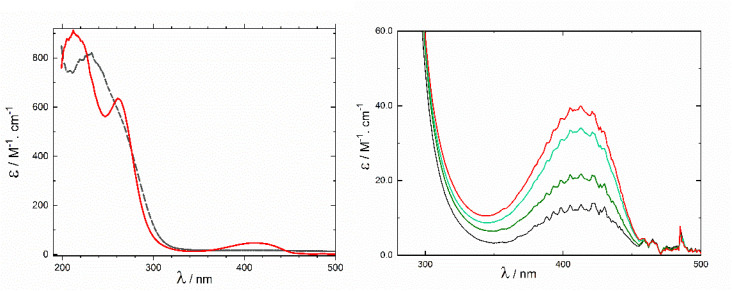
Left: change of UV-Vis spectrum of 4.14 mM 1 (dashed curve) upon addition of metallic Hg to the unstirred solution in acetonitrile (red curve, 80 minutes). Right: detail of spectral changes in time after addition of Hg. Spectra were recorded 30 (black), 40, 60, and 80 min (red) after the addition of Hg. Spectra were measured in a 1 cm cuvette and the absorbance was divided by the concentration.

This Section will follow the time dependence of the rate of the cleavage of C–Br bonds. UV-Vis spectroscopy shows that compound 1 is characterized by a single band at 219 nm (dashed line in (Fig. 3 left). The calculation of the spectrum corresponds with experimental data (Fig. 3, left, dashed line, Table S1). Experimental conditions for all UV-Vis spectroscopic measurements emerged from these preliminary observations. Solvent and the sample were meticulously dried and transferred to optical cuvettes.

**Fig. 4:**
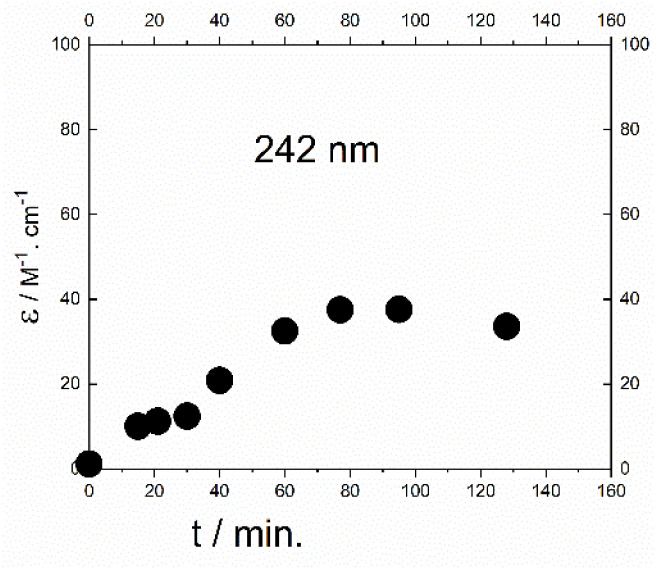
The time dependence of absorbance of 2 at 412 nm. Spectra were measured in a 1 cm cuvette and the absorbance was divided by the concentration.

**Scheme 3:**
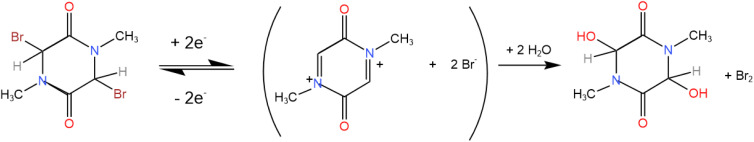
After a prolonged time, compound 2 hydrates the piperazine double bonds, releases bromine, and loses its intended activity.

The solution of a blank yields the only absorption at 230 nm (Fig. 3 dashed curve). Mercury addition changes the spectrum over 80 minutes, forming new bands at 215, 260 nm and 412 nm (Fig. 3 red curve). Calculated transitions for compound 2 are in agreement with the experimental spectrum. Details of the time dependence are in Fig. 3, right panel, and in Fig. 4. The absorption band at 412 nm increases for about 1 hour. Compound 2 is not very stable. The lifetime depends on its concentration and is critically influenced by the presence of traces of moisture. Over a longer time, compound 2 undergoes the hydration of double bonds in the piperazine ring, changing two Br^−^ anions into Br_2_ during the process (Scheme 3).

Compound 2 is stable for about two hours (Fig. 3 and 4). It adds traces of water to the double bonds of the piperazine ring. Calculations of the spectrum of hydroxy derivate using the DFT method indicated two bands at 234 nm and 244 nm, which is consistent with observed experimental bands at 260 nm. Formation of hydroxy-adduct was confirmed by NMR, UV-vis spectra (Table S1). Other products formed by the protonation of oxygen were also considered; however, none of them was detected. Calculated possible side products are listed in Table S1.

### 3.3 NMR spectroscopy

**Fig. 5:**
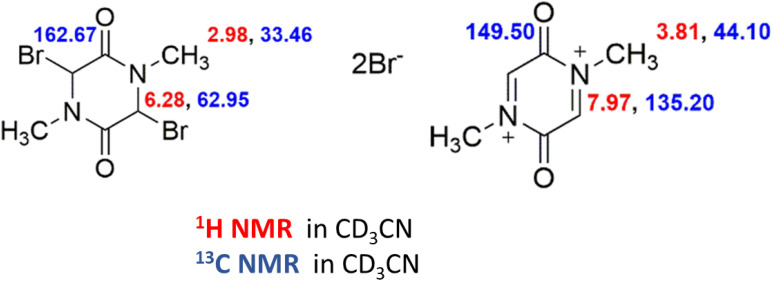
Shift of atoms in ppm.

This Section will correlate hydrogen and carbon isotopes for the presence of 2. Preparation of samples for NMR spectra is rather tricky. The reason is a very high sensitivity for traces of moisture in a carefully dried solvent. The drying procedure is given in the Experimental. Results were related to the line of CH_3_CN (Fig. 5).

**Fig. 6:**
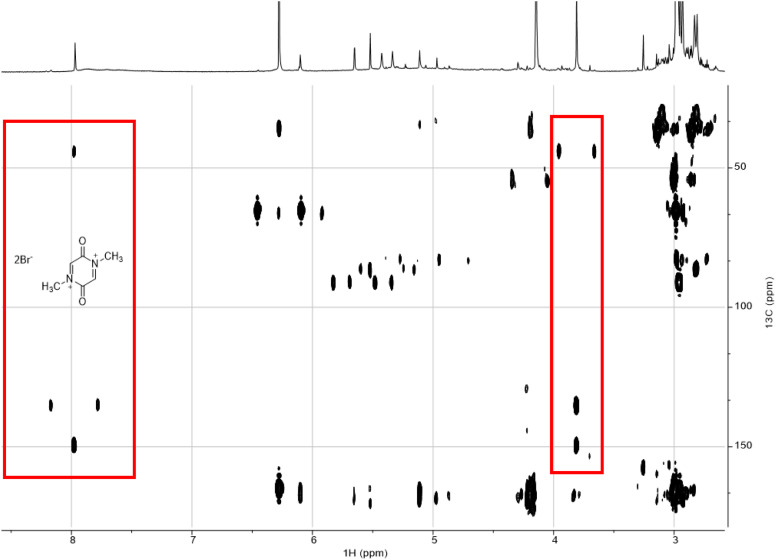
^1^H and ^13^C resonances were assigned using H, C-HMBC (heteronuclear multiple bond correlation) technique.

Two red quadrats in Fig. 6 mark the area that corresponds to the product **2**. All other resonances correspond to side products.

### 3.4 Mass spectrometry

**Fig. 7:**
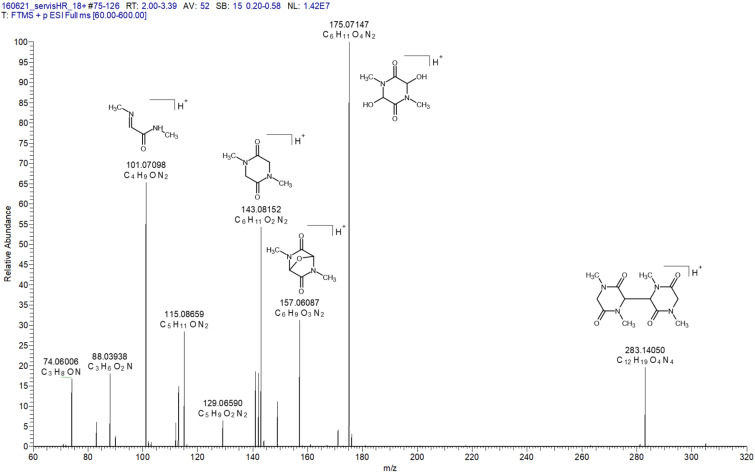
High-resolution ESI^+^ mass spectrum measured after the addition of mercury to the solution of 1 in acetonitrile. Suggested structures are provided for the abundant peaks.

The acetonitrile solution of 1, treated with metallic mercury under argon, was loop-injected into the solvent flowing into the electrospray ion source. A mixture of methanol and water (Fig. 7 and Table S2), as well as acetonitrile, was used as the carrier liquid. The group of peaks *m*/*z* 141.06599 (C_6_H_9_O_2_N_2_^+^), *m*/*z* 142.07376 (C_6_H_10_O_2_N_2_^+^), and *m*/*z* 143.08152 (C_6_H_11_O_2_N_2_^+^) suggests the formation of 2. The first peak from this group is consistent with the protonated 2 in its bi-radical state, or an intramolecular radical recombination product of the bi-radical. The other two can be explained by protonated products of radical recombination of 2 with one and two hydrogen atoms, respectively. The structures suggested for C_6_H_9_O_2_N_2_^+^ and C_6_H_10_O_2_N_2_^+^ are shown in Fig. 9. Radical recombination also leads to the formation of a dimer, whose protonated form provides *m*/*z* 283.14050 (C_12_H_19_O_4_N_4_^+^). Recombination of radical species prevents their detection by EPR spectroscopy. The spectrum base peak *m*/*z* 175.07147 (C_6_H_11_O_4_N_2_^+^) is due to the addition of water traces to the double-bonds. The other two signals, *m*/*z* = 101,07098 (C_4_H_9_ON_2_^+^) and *m*/*z* = 115.08659 (C_4_H_9_ON_2_^+^), indicate the opening of the heterocyclic ring. The list of other peaks is given in Table S2.

**Fig. 8:**
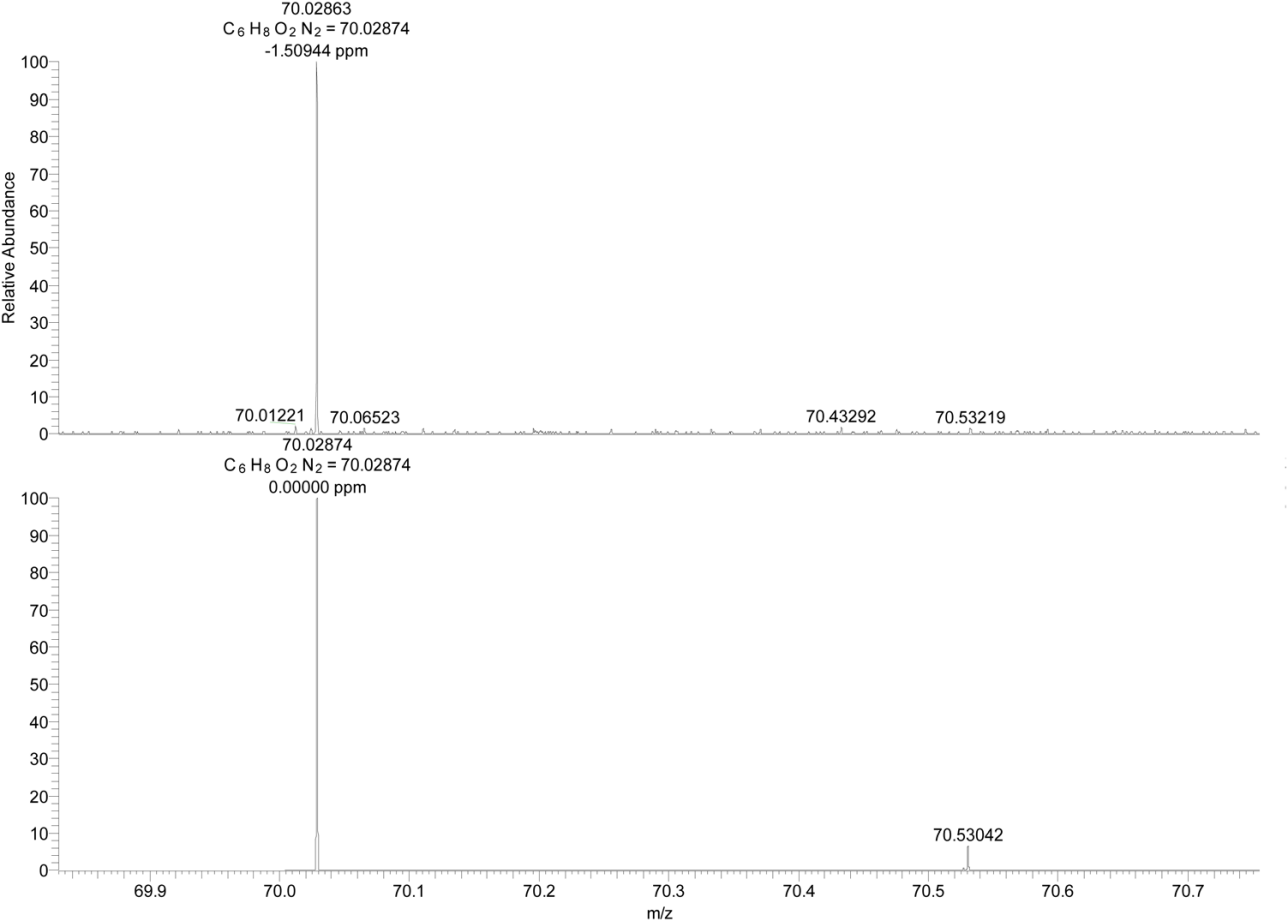
Section of the ESI^+^ mass spectrum of a solution of 1 treated with metallic mercury under argon (upper spectrum) and the simulated spectrum of the doubly charged compound 2 (lower spectrum). The sample was introduced *via* loop injection into acetonitrile flowing into the ion source.

Upon close inspection of the low *m/z* region of the spectrum, di-cation 2 appears slightly above the noise level, particularly when acetonitrile is used as the carrier liquid (Fig. 8 and S2). The experimental spectrum (upper line) fits with the value of the simulated spectrum (lower line in Fig. 8). We can conclude that the compound 2 was indeed detected. Low yield might be caused by technical problems during the transfer from electrochemical cell to the spectrometer.

**Fig. 9:**
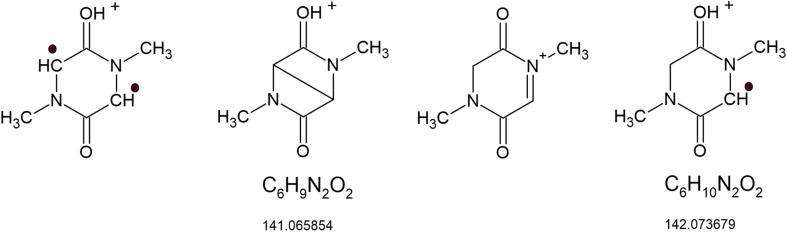
Suggested structures of the reaction products of 1 with mercury.

## 4 Conclusion

Compound 1 (3,6-dibromo-1,4-dimetnyl-piperazine-2,5-dione) suggested as a precursor for SF can easily cleave two C–Br bonds by electrochemical means or by adding mercury to the solution of 1. Electron transfer is coupled with a strong adsorption of 1 on the mercury and promotes Hg–Br bonds breaking. This results in the formation of a di-anion 2 and the adsorbed Hg_2_Br_2_ layer. The reversible redox potential of 2 is −0.15 V. The electrode material (glassy carbon) changes the electron transfer mechanism. Using Hg simplifies considerably the preparation protocol for this type of heterocyclic compounds. However, product 2 is highly reactive and we were not able to prepare its crystalline form. High reactivity of 2 makes the compound less practical for application.

## Author contributions

P. Dron and M. Dudič investigation. R. Pohl investigation, NMR. J. Cvačka investigation, MS. F. Vavrek. data curation. L. Pospíšil methodology electrochemistry, writing – review editing.

## Conflicts of interest

The authors declare that they have no known competing financial interests or personal relationships that could have appeared to influence the work reported in this paper.

## Supplementary Material

RA-015-D5RA07891A-s001

## Data Availability

Data for this article, including polarography, cyclic voltammetry, UV-Vis spectra, NMR and MS spectra are available at Zenodo repository at https://doi.org/10.5281/zenodo.17866310. Supplementary information (SI) is available. See DOI: https://doi.org/10.1039/d5ra07891a.
