# Supplementary material for: Heterocyclic biradicaloid for singlet fission: cleavage of bromine atoms from precursor 3,6-dibromo-1,4-dimethyl-piperazine-2,5-dione
Source: RSC Adv. 2025 Dec 16;15(58):50179–85. doi: 10.1039/d5ra07891a (PMC12706457; doi:10.1039/d5ra07891a)
Supplement: RA-015-D5RA07891A-s001 [file RA-015-D5RA07891A-s001.pdf]

Supplementary Information (SI) for RSC Advances.
This journal is © The Royal Society of Chemistry 2025

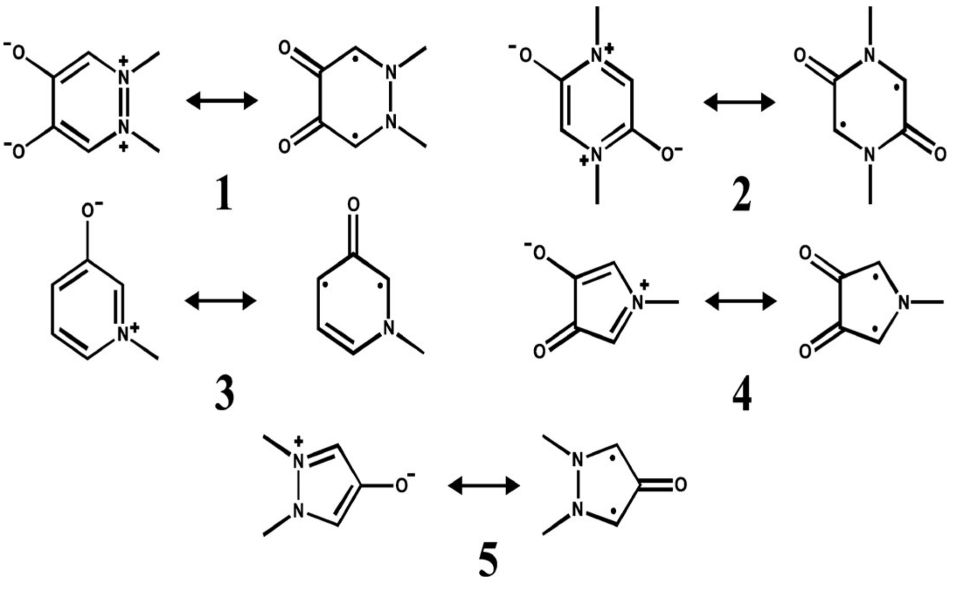

Figure S1 List of five other compounds predicted for the future study. Numeber **2** was used in this paper.

opoundt

Quantum chemical calculations

DFT method using B3LYP functional and 6-31+G* basis set as implemented in Spartan´18 was used for calculation of UV-vis spectra.

Table S1

Compound	DFT Calculated Spectra
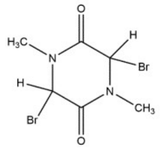 1	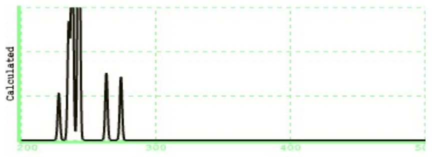
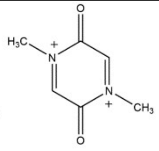 2	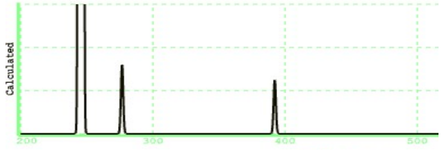

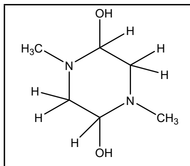	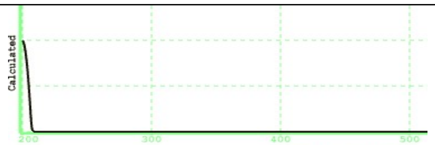
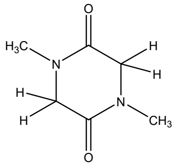	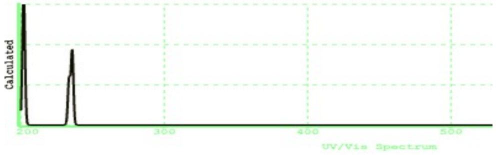
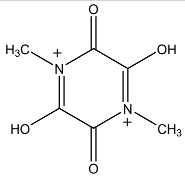	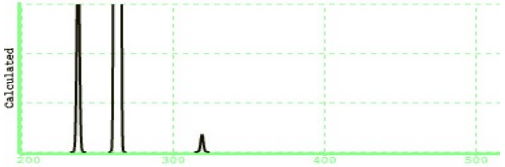
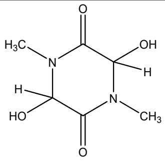	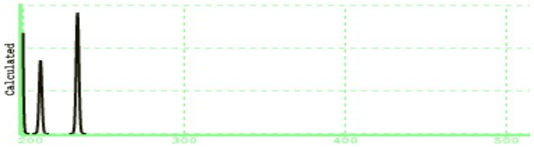
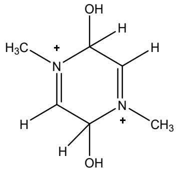	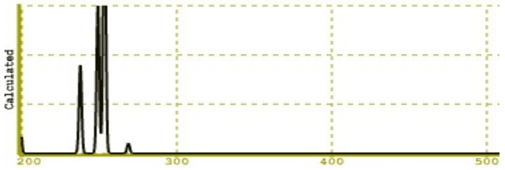
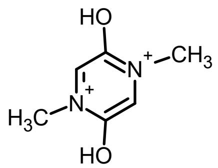	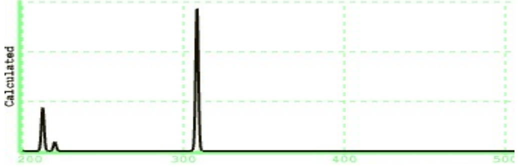
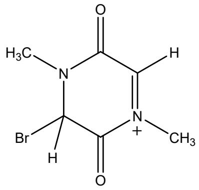	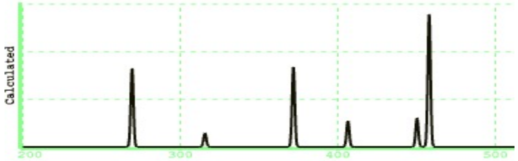
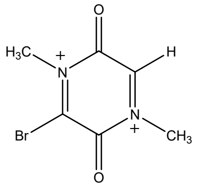	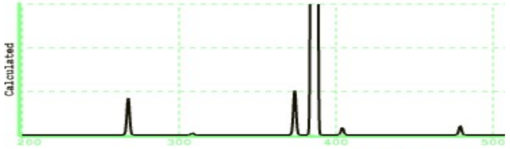
λ	200 300 400 nm

MS experiental data

Table S2

**Formula**	**m/z**
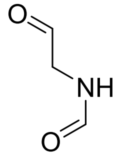<br>Chemical Formula: $C_3H_5NO_2$<br>m/z: 87.0320 (100.0%), 88.0354 (3.2%)	87<br>88
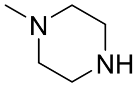<br>Chemical Formula: $C_5H_{12}N_2$<br>m/z: 100.1000 (100.0%), 101.1034 (5.4%)	100.1
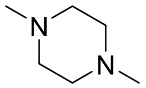<br>Chemical Formula: $C_6H_{14}N_2$<br>m/z: 114.1157 (100.0%), 115.1191 (6.5%)	114.1<br>115.1
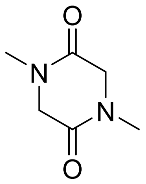<br>Chemical Formula: $C_6H_{10}N_2O_2$<br>m/z: 142.0742 (100.0%), 143.0776 (6.5%)	142<br>143
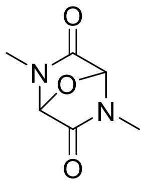<br>Chemical Formula: $C_6H_8N_2O_3$<br>m/z: 156.0535 (100.0%), 157.0568 (6.5%)	157<br>157

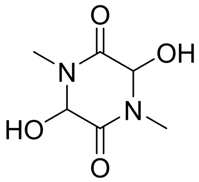<br>Chemical Formula: $C_6H_{10}N_2O_4$<br>m/z: 174.0641 (100.0%), 175.0674 (6.5%)	174<br>175
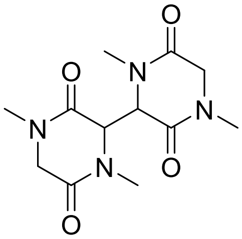<br>Chemical Formula: C12H18N4O4<br>m/z: 282.1328 (100.0%), 283.1362 (13.0%), 283.1298 (1.5%)	282.13<br>283.14<br>283.23

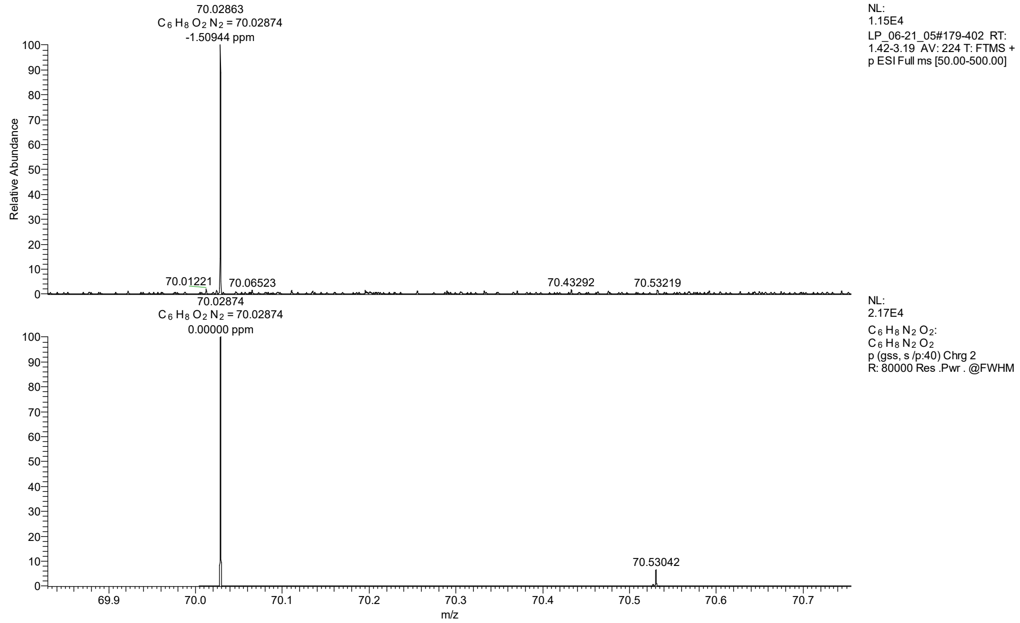

Figure S2 Section of the ESI+ mass spectrum of a solution of **1** treated with metallic mercury under argon (upper spectrum) and the simulated spectrum of the doubly charged compound **2** (lower spectrum). The sample was introduced via loop injection into acetonitrile flowing into the ion source.
